# Primary care doctors in acute call-outs to severe trauma incidents in Norway – associations with factors related to patients and doctors

**DOI:** 10.1080/02813432.2023.2216235

**Published:** 2023-05-31

**Authors:** Kristian Rikstad Myklevoll, Erik Zakariassen, Tone Morken, Valborg Baste, Jesper Blinkenberg, Gunnar Tschudi Bondevik

**Affiliations:** a Section for General Practice, Department of Global Public Health and Primary Care, University of Bergen, Norway; b National Centre for Emergency Primary Health Care, NORCE Norwegian Research Centre, Bergen, Norway

**Keywords:** Prehospital emergency care, trauma care systems, primary care doctor, out-of-hours care, casualty clinic, Norway

## Abstract

**Objective:**

Severe trauma patients need immediate prehospital intervention and transfer to a specialised trauma hospital. In Norway, primary care doctors (PCDs) are an integrated part of the prehospital trauma care. The aim of this study was to investigate the degree to which PCDs were involved in prehospital care of severe trauma patients and how factors related to patients and doctors were associated with call-outs to these incidents.

**Design:**

This was a registry-based study in Norway on severe trauma patients with acute hospital admission during the period 2012–2018.

**Setting:**

Data was obtained from three Norwegian official registries.

**Subjects:**

By linking the registries, we studied the actions taken by the PCDs, whether they called out to severe trauma incidents.

**Main outcome measures:**

In multivariable regression models, we investigated whether factors related to the PCDs (age, sex, specialisation in general practice (GP)) and patients (age, sex, duration of hospital stay, type of injury) were associated with call-outs.

**Results:**

Out of 4342 severe trauma incidents, PCDs had documented involvement in 1683 (39%) and called out to 644 (15%). Increased proportions of PCD call-outs to severe trauma incidents were significantly associated with lower age of PCD, being a GP specialist, lower patient age, being a male patient, increased length of hospital stay and injuries to the head and the neck.

**Conclusions:**

PCDs called out to a relatively low proportion of severe trauma patients. Several factors related to patients and doctors were associated with call-outs to severe trauma incidents in Norway.

## Key points

Factors related to doctors and patients affect the Primary Care Doctor’s (PCD’s) decision to call out to severe trauma incidents.

PCDs were involved in 39% out of 4342 severe trauma incidents and called out to 15%.

Increased proportion of PCD call-outs to severe trauma incidents was significantly associated with lower age of the PCD and being a GP specialist.

Lower patient age, being a male patient, and injury to the head and the neck increased the likelihood of PCD call-outs.

## Introduction

A patient with severe trauma needs immediate prehospital intervention and transfer to a specialised trauma hospital for definitive care [1]. In Norway, like in most Western countries, a comprehensive trauma system in accordance with the “Trauma chain of survival” has been described and agreed upon [2,3]. The chain of survival includes early first aid, life support, advanced therapy and rehabilitation.

In Norway, the prehospital emergency services are shared between the primary and the secondary health care services. The municipalities are responsible for emergency primary health care, with primary care doctors (PCDs) on-call and casualty clinics open 24 h a day [4]. In the present study, a PCD is defined as a doctor on-call, working as a general practitioner (GP) or in an out-of-hours (OOH) casualty clinic. The secondary health care services are organised by the State through regional health authorities. They are responsible for hospitals, ambulance services, helicopter emergency medical services (HEMS) and the emergency medical communication centres (EMCCs) [4].

Inhabitants are supposed to call 113 to the EMCC if they suspect a life-threatening medical problem. If the triage concludes with a presumably life-threatening situation, the EMCC should send an alarm to the PCD on-call and despatch the ambulance as an acute response. The PCD on-call has to decide whether to call out to the scene of the accident. If needed, the alarm is also sent to the HEMS [4].

Although HEMS physicians have the best competence in treating severe trauma patients, HEMS must regularly reject missions due to weather, concurrency, technical conditions or flight regulations [5,6]. The PCDs and ambulance services could therefore be the only emergency healthcare staff available for call-outs. They have an important role in treating severe trauma patients together in a prehospital setting and are considered the backbone of prehospital care in Norway [7–9].

Ambulance staff have two years education in upper secondary school and two years of apprenticeship [10,11]. Ambulances are despatched by the EMCCs and cannot decide themselves whether or not to call out. On the other hand, the PCD is supposed to call out when needed and must choose between a call-out to a possible severely injured patient or stay at the casualty clinic to treat other patients [12]. In a previous study, it was found that PCDs in Norway were alarmed in 47% of all acute alarms from the EMCCs [13]. Of these, PCDs called out to 42%.

A PCD at the scene of an accident could reduce both under- and over-triage [14]. A patient with no sign of severe injury after triage may not need admission to a trauma hospital. These patients could be given adequate care by the PCD [3]. This would avoid delays in care and lessen the risk of over-treatment. Less over-triage would also ease the burden on hospitals. However, a systematic review found no studies on the effect of having a PCD at the scene of an accident [15]. The PCD needs to master emergency treatment both in a team setting together with ambulance staff [16,17] and alone as the first healthcare responder attending to a trauma patient. Since 2015, a new regulation has demanded emergency work experience and specific training in emergency medicine for qualification as an OOH doctor in Norway. If needed, PCDs without these qualifications have to consult an experienced PCD [4].

In an earlier Norwegian focus group study, PCDs perceived their role as an important part of prehospital emergency care [18]. It was argued that it was sometimes difficult to decide when to call out due to limited information in the primary message from the EMCC. More knowledge about factors related to the PCD call-out to suspected severe trauma would be useful in planning prehospital emergency care.

The aim of the present study was to investigate the degree to which PCDs were involved in prehospital care of severe trauma patients and how factors related to patients and doctors were associated with call-outs to these incidents.

## Material and methods

This was a registry-based study for which data was obtained from all acute admissions to Norwegian hospitals in the seven-year period from 2012–2018 from the Control and Payment of Reimbursement to Health Service Providers database (KUHR), the Norwegian Patient Registry (NPR) and Statistics Norway (SSB).

After a patient contact, Norwegian PCDs make a claim to the KUHR. Such a claim contains patient information including the patient's national identification number, contact date and time and type of contact (clinic consultation, telephone contact or home visit/call-out).

After hospital admission, the information is registered in the NPR. This registry contains information about patients’ national identification numbers, date and time of admission and the degree of urgency. The consultant at the hospital has to register an International Statistical Classification of Diseases and Related Health Problems Version 10 (ICD-10) code. Chapter 19, which is also referred to as block S-T is diseases classified as Injuries, Poisoning and Certain Other Consequences of External Causes. The hospital makes Diagnosis-Related Group (DRG) codes, an internationally validated code system based on the ICD-10 code and other patient information. A certain code is expected to have similar hospital resource use and the Government Use it to pay the hospitals. The DRG code is stored in the NPR. SSB created a pseudo-anonymised identification number for this study. This number replaced the national identification number in the KUHR and NPR databases and made it possible to link data from both registries without revealing patients’ identities.

In the present study, we assume that a PCD contact less than 24 h before hospital admission is the specific PCD involvement in the severe trauma incident. We use this to link PCD contacts in the KUHR to corresponding hospital admissions in the NPR. In a previous study, a distinct connection was shown between hospital admissions and PCD consultations less than 24 h before the admission [19,20]. In our study, some of the KUHR account cards were written after the time of the hospital admission. Since all these patients were admitted for more than 24 h, it is likely that the PCDs have carried out the patient assessment and treatment prior to the admission. Later, when having time, the PCDs have written the KHUR account card. The time on the card is registered automatically, without changing the time back to the time of the incident. For that reason, we included PCD consultations documented less than 24 h before - and 12 h after - the hospital admission.

We identified severe trauma incidents likely to have resulted in an acute response alarm to PCD and ambulance services by including the following all four selected DRG codes: Craniotomy at significant for multi-trauma, Major surgery hip/femur and replantation, Operations at significant multi-trauma and Significant multi-trauma (DRG codes 484-487). All these patients were discharged from the hospital with severe trauma as the main diagnosis. It is reasonable to assume that these severe trauma incidents led to acute response alarms from the EMCCs to on-call PCDs and ambulance services. Therefore, patients with these four DRG codes were included in our material.

We recorded the PCD sex (male, female), PCD age in years (≤35, 36–45, 46–55, ≥56), specialist training (GP specialist or not), patient sex (male, female), patient age in years (≤20, 21–40, 41–60, 61–80, ≥81), discharge diagnosis according to anatomic site of injury (head and neck, chest area, abdomen and pelvic area and extremities), and duration of hospital stay in days (≤2, 3–5, 6–10, ≥11).

Descriptive statistics were conducted with frequency distributions and mean values. To study the associations between PCD and patient characteristics and the likelihood of PCD call-out to severe trauma patients, generalised linear models were used to estimate relative risk (RR) with 95% confidence interval (CI). Both crude and adjusted analyses are presented. The analyses were adjusted for all seven independent variables in the same model. Statistical significance was set at α = 0.05 and the data was analysed by the statistical program, Stata 16.1. (StataCorp. 2019. Stata Statistical Software: Release 16. College Station, TX: StataCorp LLC.)

## Results

**Figure 1.**
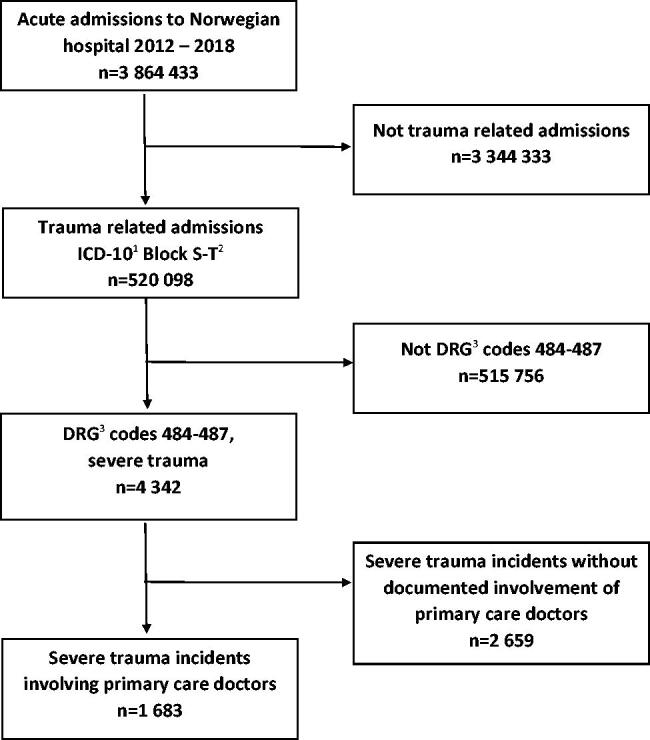
Trauma incidents in Norway, hospital admissions and primary care doctor involvement, 2012–2018. ^1^International Statistical Classification of Diseases and Related Health Problems version 10 (ICD-10). ^2^Block S-T, (Chapter 19) of ICD-10 are diseases classified as Injuries, Poisoning and Certain Other Consequences of External Causes. ^3^Hospitals make Diagnosis-Related Group (DRG) codes, an international validated code system based on the ICD-10 code and other patient information.

The total number of acute hospital admissions in Norway in the period from 2012–2018 was 3864 433. Of these, 520 098 admissions were due to injury with ICD-10 codes from block S-T. There were 4342 acute admissions due to the four DRG codes included for severe trauma with the following distribution: Craniotomy at significant multi-trauma (*n* = 193), Major surgery hip/femur and replantation (*n* = 277), Operations at significant multi-trauma (*n* = 1513), and Significant multi-trauma (*n* = 2359). PCDs were involved in 1683 (39%) of these trauma incidents (Figure 1). There was no documented PCD involvement in the remaining 2659 incidents (61%) that were handled by ambulance services and hospitals.

Of the 1683 severe trauma incidents that involved PCDs, they called out to 644 (38%), 318 (19%) were handled by telephone contact with the PCD and 721 (43%) by a clinic consultation with the PCD at the casualty clinic after the injured patient had been taken there (Table 1). Overall, PCDs called out to 15% (644 out of 4342) of severe trauma incidents.

**Table 1.** Contact type in severe trauma incidents involving primary care doctors, Norway, 2012-2018.

Contact type	n	%
Call-out	644	38.3
Clinic consultation	721	42.8
Telephone contact	318	18.9
Total	1683	100

The proportion of PCD call-outs decreased with increasing age of the PCD (Table 2). The adjusted relative risk (RR) (95%CI) for the PCD age groups 36–45, 46–55 and ≥ 56 years was 0.85 (0.73–0.98), 0.72 (0.59–0.89) and 0.60 (0.48–0.75) respectively, compared to the reference PCD age category ≤ 35 years. The GP specialist category had an increased proportion of call-outs, adjusted RR 1.34 (1.15–1.56) compared to PCDs without this specialty.

**Table 2.** Primary care doctor (PCD) call-outs to severe trauma patients, Norway, 2012–2018.

	PCD call-out to severe trauma patients						
Variable	N^a^	N^b^	%	RR_crude_	95%CI	RR_adj_^c^	95% CI
Total	1683	644	38.3				
PCD Age (years)							
≤35	495	225	45.5	1.00		1.00	
36-45	417	172	41.3	0.91	0.78–1.05	0.85	0.73–0.98
46-55	216	77	35.7	0.78	0.64–0.96	0.72	0.59–0.89
≥56	227	70	30.8	0.68	0.55–0.84	0.60	0.48–0.75
PCD Sex							
Male	934	371	39.7	1.00		1.00	
Female	421	173	41.0	1.03	0.90–1.19	0.95	0.84–1.09
GP specialist							
No	1030	402	39.3	1.00		1.00	
Yes	369	157	42.6	1.12	0.97–1.29	1.34	1.15–1.56
Patient Age (years)							
≤20	185	102	55.1	1.00		1.00	
21–40	254	134	52.8	0.96	0.80–1.14	0.95	0.79–1.14
41–60	359	164	45.7	0.83	0.69–0.98	0.89	0.74–1.06
61–80	466	156	33.5	0.61	0.51–0.73	0.59	0.49–0.72
≥81	419	88	21.0	0.38	0.30–0.48	0.43	0.34–0.55
Patient Sex							
Male	1017	441	43.4	1.00		1.00	
Female	666	203	30.5	0.70	0.61–0.80	0.83	0.72–0.96
Discharge Diagnosis (ICD-10) Injuries to the							
Extremities	502	172	34.3	1.00		1.00	
Abdomen and pelvic area	484	159	32.9	0.96	0.80–1.14	0.80	0.67–0.97
Chest area	362	152	42.0	1.23	1.03–1.45	1.14	0.96–1.35
Head and neck	335	161	48.0	1.40	1.19–1.65	1.30	1.10–1.54
Duration of hospital stay (days)							
≤2	399	139	34.8	1.00		1.00	
3–5	439	140	31.9	0.92	0.76–1.11	0.94	0.77–1.14
6–10	435	149	34.2	0.98	0.82–1.19	1.08	0.89–1.31
≥11	410	216	52.7	1.51	1.29–1.78	1.53	1.30–1.81

^a^N = number of severe trauma patients with PCD involvement.

^b^n = number of severe trauma patients with PCD call-out.

^c^Adjusted relative risk (RR) obtained from generalized linear model adjusted for the variables included in this table, using the first category of each variable as reference category.

GP factors: 1355 observations. Patient factors: 1683 observations.

The proportion of PCD call-outs decreased significantly with increasing patient age >60 years. For the patient age groups 61-80 and ≥81 years, RR of 0.59 (0.49–0.72) and 0.43 (0.34–0.55) respectively were found compared to the reference patient age category ≤ 20 years. Being a female patient was associated with a significantly lower proportion of PCD call-outs to suspected severe trauma (RR = 0.83 (0.72–0.96)) compared to being a male patient. The presence of head and neck injuries increased the proportion of PCD call-outs compared to the reference category, extremity injuries (RR = 1.30 (1.10–1.54)). The proportion of PCD call-outs to patients with abdominal and pelvic injuries was significantly lower with RR 0.80 (0.67–0.97) compared to the reference category. A severely injured patient with a hospital stay ≥ 11 days was associated with a higher proportion (53%) of a prior PCD call-out, compared to patients with a hospital stay ≤ 2 days.

## Discussion

### Statement of principal findings

PCDs were involved in 1683 (39%) out of 4342 severe trauma incidents and called out to 644 (15%). There was no documented PCD involvement in the remaining 2 659 incidents (61%) that were handled by ambulance services and hospitals. Increased proportion of PCD call-outs to severe trauma incidents was significantly associated with lower age of the PCD, being a GP specialist, lower patient age, being a male patient, increased length of hospital stay and injury to the head and the neck.

### Strengths and weaknesses of the study

This was a registry-based study with highly reliable data, including all acute admissions to all somatic Norwegian hospitals in a seven-year period from 2012 to 2018. Thus, we avoided potential selection bias of sub-groups for further analysis. Using these registries, we were able to measure the degree of documented PCD involvement in prehospital care for severe trauma patients, as well as identify whether factors related to the patients and doctors were associated with call-out.

There was no documented PCD involvement in 2659 (61%) of the 4342 severe trauma incidents. This could be for several reasons. According to the EMCC guidelines, it should be reasonable to assume that included severe trauma incidents led to acute response alarms to on-call PCDs and ambulance services. However, it is not possible to confirm using our data whether an alarm was actually sent from EMCC or whether it was only sent to ambulance services and not to the PCD. It is also possible that some of the trauma incidents managed by PCDs through clinic consultation or telephone contact had less urgent initial presentation, thus not through the EMCC system. Further, it may be possible that PCDs were alarmed and involved in some of the 2659 severe trauma incidents but that the call-outs, telephone contacts and clinic consultations were not documented in the KUHR. This is a limitation of the study that also leads to uncertainty regarding the actual call-out rate to severe trauma incidents. Future studies should investigate the degree to which EMCCs send alarms to PCDs in severe trauma incidents, as well as the extent of not documenting the PCD involvement in these acute situations.

Most acute admissions with ICD-10 codes in blocks S and T are not due to severe trauma but minor injuries. In our study, we wanted to include a sample of severe trauma incidents only where it was likely that an acute response alarm had been sent from EMCC to the on-call PCD and ambulance services. To ensure that, we included significant multi-trauma patients with DRG codes 484–487, incidents for which it could be expected that a PCD had received an alarm from the EMCC and could contribute substantially at the accident scene. As many severely injured patients are not classified in one of these four DRG codes, it meant that they were not included in our study. This is a limitation, as a larger sample would have strengthened the statistical power of the study with increased opportunities for subgroup analyzes. We still think it is likely that our results would have been similar due to a relatively large data set covering several years. However, it was essential to not include patients with minor injuries, which would have been the case if we had included patients based on the ICD-10 codes.

### Findings in relation to other studies

Overall, PCDs called out to only 15% of the 4342 severe trauma incidents. This is somewhat lower than found in a Norwegian study investigating all acute responses in the catchment area of three EMCCs [13]. In that study, PCDs were alarmed in 47% of acute responses and called out in 42% of these, corresponding to an overall call-out rate of 20%. The previous study is from 2010, a time when the guidelines for when EMCCs should alarm PCDs were less clear than today. Further, the study investigated alarms for all types of acute response, including both diseases and injuries. A third point is that our study included patients from Oslo with many patients and low degree of PCD involvement, due to short distances to ambulance and hospital services. This may explain the lower overall call-out rate in our study. A Norwegian study from 2019 found an overall PCD involvement in 64% of acute hospital admissions [19,20]. PCDs were involved in 58% of acute admissions for acute myocardial infarction, 58% of cerebral infarctions, 59% of intracranial injuries and 50-57% of admissions for different types of non-severe extremity fractures. These proportions are higher than the 39% found in our study and may indicate that PCDs are more involved in incidents where protocols for direct admission to hospital by the ambulance services are less relevant.

A PCD call-out rate of only 15% to severely injured patients is not in line with the National Trauma Plan. There should be organisational changes to enable PCDs to call out more frequently. There also is a need for clearer guidelines for when to call out to certain acute medical conditions, including suspected severe trauma. As ambulance services and PCDs are the backbone of the prehospital trauma care in Norway [7], EMCCs should always alert both the PCD and ambulance in the event of a severe incident, as required by the guidelines in emergency medicine regulations [4]. There is uncertainty about the possible effects of having a PCD at the scene of accident [15], and we could argue that the National Trauma Plan is not up to date, by involving the PCD. However, emergency trauma situations are initially often unclear, there could be several injured, more or less severe. Further, the PCD on-call in rural areas may have shorter travel time to the accident scene compared to the HEMS, in situations where a doctor is needed. Finally, the HEMS must regularly reject missions due to weather, concurrency, technical conditions or flight regulations. Based on this, we think it is wise to continue including PCD involvement in the Norwegian national trauma plan, and that there is reason to state that the low call-out rate in our study is problematic.

We observed that the proportion of PCD call-outs to severe trauma patients decreased significantly with increasing age of the PCD. This could be due to many factors, such as time of medical training, work experience, workload, work endurance and PCD health. Both the healthcare system and health professional training have changed over time. Three studies investigating the variation in referral frequency among GPs in OOH care found that older PCDs referred less patients to hospital compared to younger PCDs [21–23]. This indicates that PCD age is an important factor affecting decisions made in the OOH setting.

The analyses showed that PCDs with a GP speciality had significantly increased proportions of call-outs to suspected severe trauma compared to PCDs without this speciality. Becoming a GP specialist in Norway requires a PCD to fulfil several requirements, including work in a casualty clinic and a compulsory course in prehospital emergency medicine, which must be repeated every five years in order to retain the GP specialist status [4]. Specialist training leads to better knowledge and skills, including prehospital trauma care. A study investigating the variation in referral frequency among PCDs in OOH care indicated that being a GP specialist was associated with less acute referrals to hospital [21]. GP specialists may call out to the scene of an accident to a higher degree to consider whether hospital admission is actually needed or whether the injured patient can be treated in primary care.

The proportion of PCD call-outs to severe trauma decreased significantly with increasing patient age > 60 years. The same pattern was observed in a Norwegian study of trauma patients older than 64 years compared to younger patients [24]. Patients older than 64 years received less treatment in both the prehospital and hospital settings, and procedures were performed with a delay in comparison to younger patients. A study from Scotland on prehospital critical care teams noted that young patients were treated more often by the teams [11]. The authors described this as a correct priority due to less high energy accidents among older people. A scoping review from 2021 observed under-triage in prehospital treatment of elderly trauma patients [25]. Cognitive disease, delirium and altered physiological parameters can sometimes make it difficult to detect signs of trauma in the elderly. Under-triage may be a possible explanation for the significantly lower proportion of PCD call-outs to older trauma patients in our study.

PCDs called out to suspected severe trauma incidents with male patients more frequently compared to female patients. The study mentioned from Scotland also noted that male patients were more often treated by prehospital critical care teams [11]. As males are generally overrepresented in accidents and particularly in high energy accidents, this was considered to be a correct priority. A Canadian study investigating outcome in common procedures noted differences due to sex discordance between surgeon and patient [26]. Female patients who underwent surgery with a male surgeon had worse outcomes. This is not directly comparable to our study but indicates that patient sex could affect outcome or type of treatment given. In our multivariable regression model, we have adjusted for patient age, duration of hospital stay and diagnosis group. Nevertheless, we still found a significant difference in PCD call-outs between male and female patients. In the Norwegian Trauma Register annual report for 2021 it is stated that male patients are admitted to trauma centres more often for all age groups under the age of 80 years [27]. Future studies should investigate whether PCDs call out more often to male patients based on information about patient sex in the primary radio message from EMCC or whether this finding may be explained by other factors.

We found that PCDs called out significant more frequently to suspected severe trauma when after hospital admission the discharge diagnosis was injury to head and neck compared to injuries to the extremities. PCDs called out less frequently when the discharge diagnosis was injury to the abdomen and pelvic area. At the time of the alarm, PCDs have limited knowledge about the patient. However, there often is information about the mechanisms of the accident and the kinds of injury the caller has observed at the scene of accident. Details regarding head and neck injuries in the primary radio message from the EMCC may increase the probability of a PCD call-out.

This study observed an increased proportion of call-outs to patients needing a hospital stay ≥ 11 days. There is a probable association between trauma severity and duration of hospital stay, which may thus act as a proxy for severity. As this was a significant finding in the adjusted analyses, further studies should investigate whether the primary radio messages from EMCCs contain information beyond patient sex, age and site of injury that may indicate the level of severity. A knowledge of high severity would help the PCD when considering the need for the call-out.

### Meaning of the study

PCDs were involved in 1683 (39%) out of 4342 severe trauma incidents and called out to 644 (15%). Ambulance services and hospitals handled 2659 incidents (61%) alone. Increased proportion of PCD call-outs to severe trauma incidents had a significant association with lower age of PCD, being a GP specialist, lower patient age, being a male patient, increased length of hospital stay and injury to the head and neck. Future studies should investigate the effect of having a PCD at the scene of an accident, the consequences of the rather low PCD call-out rate to severely injured patients and possible reasons for the patient sex difference. In addition, we recommend clearer guidelines regarding which trauma incidents need call-out by the PCD as well as ambulance services. This knowledge would be useful for planning and organising prehospital care for severely injured patients.

## Ethical approval and consent to participate

Permission for this study has been obtained from the Regional Committee for Medical and Health Research Ethics (REC) (30.01.2014 reference number 2013/2344/REK Vest) and the Norwegian Data Protection Authority has granted a licence to process personal data for research purposes (15.09.2014) (reference number 14/0322-9/CGN). The data is obtained from registers that all have as one of their tasks to contribute to research, or professional management and development. An assessment of privacy consequences (DPIA) has been prepared for the project in collaboration with NORCE's privacy representative. In the project, discharge diagnoses and time of hospitalizations will be key data. This is sensitive information about the individual, which is covered by the duty of confidentiality. However, in the research dataset, the names are removed, and the personal identification number is replaced with a serial number. The sensitive data will therefore not be possible to link to a person. It will be ensured that the units examined are not so small that persons can be identified based on findings (“pathway identification”). In this way, the risk of sensitive data about individuals going astray will be minimal. The data is stored and analysed on a secure server. The PhD project will be based on data form health registries collected from EMCC, GPs on-call, ambulance services and hospitals. The data includes information regarding acute medical conditions. Details regarding the medical conditions of the patients will be available for one of the researchers (the PhD candidate). Information that may possibly identify patients will only be available for this researcher. He is responsible for not including information that may identify patients in the final data set. The final anonymous data set will be available for the research group. The study will be conducted in compliance with the ethical guidelines of the Helsinki Declaration.
